# Mito-metformin protects against mitochondrial dysfunction and dopaminergic neuronal degeneration by activating upstream PKD1 signaling in cell culture and MitoPark animal models of Parkinson’s disease

**DOI:** 10.3389/fnins.2024.1356703

**Published:** 2024-02-21

**Authors:** Muhammet Ay, Adhithiya Charli, Monica Langley, Ahyoung Jang, Piyush Padhi, Huajun Jin, Vellareddy Anantharam, Balaraman Kalyanaraman, Arthi Kanthasamy, Anumantha G. Kanthasamy

**Affiliations:** ^1^Parkinson’s Disorder Research Laboratory, Department of Biomedical Sciences, Iowa Center for Advanced Neurotoxicology, Iowa State University, Ames, IA, United States; ^2^Department of Physiology and Pharmacology, Isakson Center for Neurological Disease Research, University of Georgia, Athens, GA, United States; ^3^Department of Biophysics, Medical College of Wisconsin, Milwaukee, WI, United States

**Keywords:** metformin, PKD1, Parkinson’s disease, mitochondria, mitochondrial biogenesis, MitoPark, neuroprotection

## Abstract

Impaired mitochondrial function and biogenesis have strongly been implicated in the pathogenesis of Parkinson’s disease (PD). Thus, identifying the key signaling mechanisms regulating mitochondrial biogenesis is crucial to developing new treatment strategies for PD. We previously reported that protein kinase D1 (PKD1) activation protects against neuronal cell death in PD models by regulating mitochondrial biogenesis. To further harness the translational drug discovery potential of targeting PKD1-mediated neuroprotective signaling, we synthesized mito-metformin (Mito-Met), a mitochondria-targeted analog derived from conjugating the anti-diabetic drug metformin with a triphenylphosphonium functional group, and then evaluated the preclinical efficacy of Mito-Met in cell culture and MitoPark animal models of PD. Mito-Met (100–300 nM) significantly activated PKD1 phosphorylation, as well as downstream Akt and AMPKα phosphorylation, more potently than metformin, in N27 dopaminergic neuronal cells. Furthermore, treatment with Mito-Met upregulated the mRNA and protein expression of mitochondrial transcription factor A (TFAM) implying that Mito-Met can promote mitochondrial biogenesis. Interestingly, Mito-Met significantly increased mitochondrial bioenergetics capacity in N27 dopaminergic cells. Mito-Met also reduced mitochondrial fragmentation induced by the Parkinsonian neurotoxicant MPP^+^ in N27 cells and protected against MPP^+^-induced TH-positive neurite loss in primary neurons. More importantly, Mito-Met treatment (10 mg/kg, oral gavage for 8 week) significantly improved motor deficits and reduced striatal dopamine depletion in MitoPark mice. Taken together, our results demonstrate that Mito-Met possesses profound neuroprotective effects in both *in vitro* and *in vivo* models of PD, suggesting that pharmacological activation of PKD1 signaling could be a novel neuroprotective translational strategy in PD and other related neurocognitive diseases.

## Introduction

As the most common neurodegenerative movement disorder, Parkinson’s disease (PD) affects millions worldwide and inflicts significant social and economic burdens on society. This disease is mainly characterized by the progressive loss of dopamin(DA)ergic neurons in the substantia nigra of the midbrain that are involved in controlling movement and motor coordination (Shulman et al., 2011; Armstrong and Okun, 2020). Although several gene mutations and environmental factors have been shown to affect the risk of PD, the exact etiology of the disease is not yet fully understood (Lai et al., 2002; Schapira and Jenner, 2011; Trinh and Farrer, 2013; Kouli et al., 2018; Choong and Mochizuki, 2023). Compelling evidence from both experimental studies and postmortem human brain tissues suggests that mitochondrial dysfunction and oxidative stress play important roles in the pathogenesis of PD (Sanders and Greenamyre, 2013; Subramaniam and Chesselet, 2013; Ryan et al., 2015; Trist et al., 2019; Malpartida et al., 2021). Interestingly, transgenic animals with either mitochondrial transcription factor A (TFAM) or mitochondrial complex I deficits in DAergic neurons recapitulate the neurobehavioral, neurochemical and neurodegenerative changes of PD (Trancikova et al., 2012; Doric and Nakamura, 2021; Henrich et al., 2023).

Protein kinase D1 (PKD1) is a key member of the PKD family that belongs to the calcium/calmodulin-dependent protein kinase (CAMK) superfamily (Manning et al., 2002). PKD1 is expressed in the brain and plays an important role in regulating several cellular functions, including Golgi function, membrane trafficking, and cell survival (Jamora et al., 1999; Prigozhina and Waterman-Storer, 2004; Storz et al., 2005). We previously demonstrated that PKD1 is highly expressed in nigral DAergic neurons and rapidly activated during oxidative stress, and its activation plays a compensatory neuroprotective function against oxidative stress-induced DAergic neuronal cell death (Asaithambi et al., 2011, 2014), while negative modulation of PKD1 signaling increased the susceptibility of DAergic neurons to oxidative stress (Ay et al., 2015).

Type 2 diabetes (T2DM) is one of the most common chronic disorders and the most common form of diabetes, affecting approximately 90% of people with diabetes (Santiago and Potashkin, 2014). There is a growing literature suggesting that T2DM is a risk factor for neurodegenerative diseases including PD (Sun et al., 2012; Santiago and Potashkin, 2013a; Hassan et al., 2020). Although the mechanism behind the association of these two diseases is currently unknown, both diseases share some similar pathogenic pathways, such as mitochondrial dysfunction, oxidative stress, endoplasmic reticulum (ER) stress, and inflammation (Sandyk, 1993; Santiago and Potashkin, 2013b; Hassan et al., 2020). Studies have also reported that diabetic animals showed increased vulnerability to Parkinsonian specific toxicants, including 6-OHDA and MPTP (Morris et al., 2010; Wang et al., 2014). Metformin is one of the most commonly used anti-diabetic drugs with a high safety profile (Dunn et al., 1995). Metformin has been shown to protect against DAergic neurodegeneration in experimental models of PD (Patil et al., 2014; Lu et al., 2016; Katila et al., 2017; El-Ghaiesh et al., 2020; Wang et al., 2020). More importantly, a cohort study revealed that the combination therapy of metformin and sulfonylurea reduced the risk of developing PD in patients with T2DM in a Taiwanese population (Wahlqvist et al., 2012). In elderly veterans with T2DM, taking metformin for more than 2 years was linked to a reduced risk of developing neurodegenerative diseases (Shi et al., 2019).

Given that mitochondrial dysfunction is an important factor in PD pathogenesis, targeting compounds into mitochondria may lead to the discovery of more specific, less toxic, and more effective neuroprotective agents for PD. Attaching the lipophilic cation triphenylphosphonium (TPP^+^) to compounds has been shown to facilitate the increased accumulation of compounds inside the mitochondria (Smith et al., 2003). In this study, we synthesized Mito-Met, a mitochondria-targeted metformin analog in conjugation with a TPP^+^ group, and then tested the neuroprotective efficacy of Mito-Met in cell culture and MitoPark mice, a transgenic mitochondrial impairment animal model of PD. Our results show that Mito-Met activates PKD1 signaling, enhances mitochondrial function, and protects against neurotoxicity in cell models of PD. Our results also reveal that Mito-Met has neuroprotective effects in the MitoPark mouse model of PD.

## Results

### Mito-Met induces activation of PKD1 and its potential downstream targets Akt and AMPK in a cell model of PD

**Figure 1:**
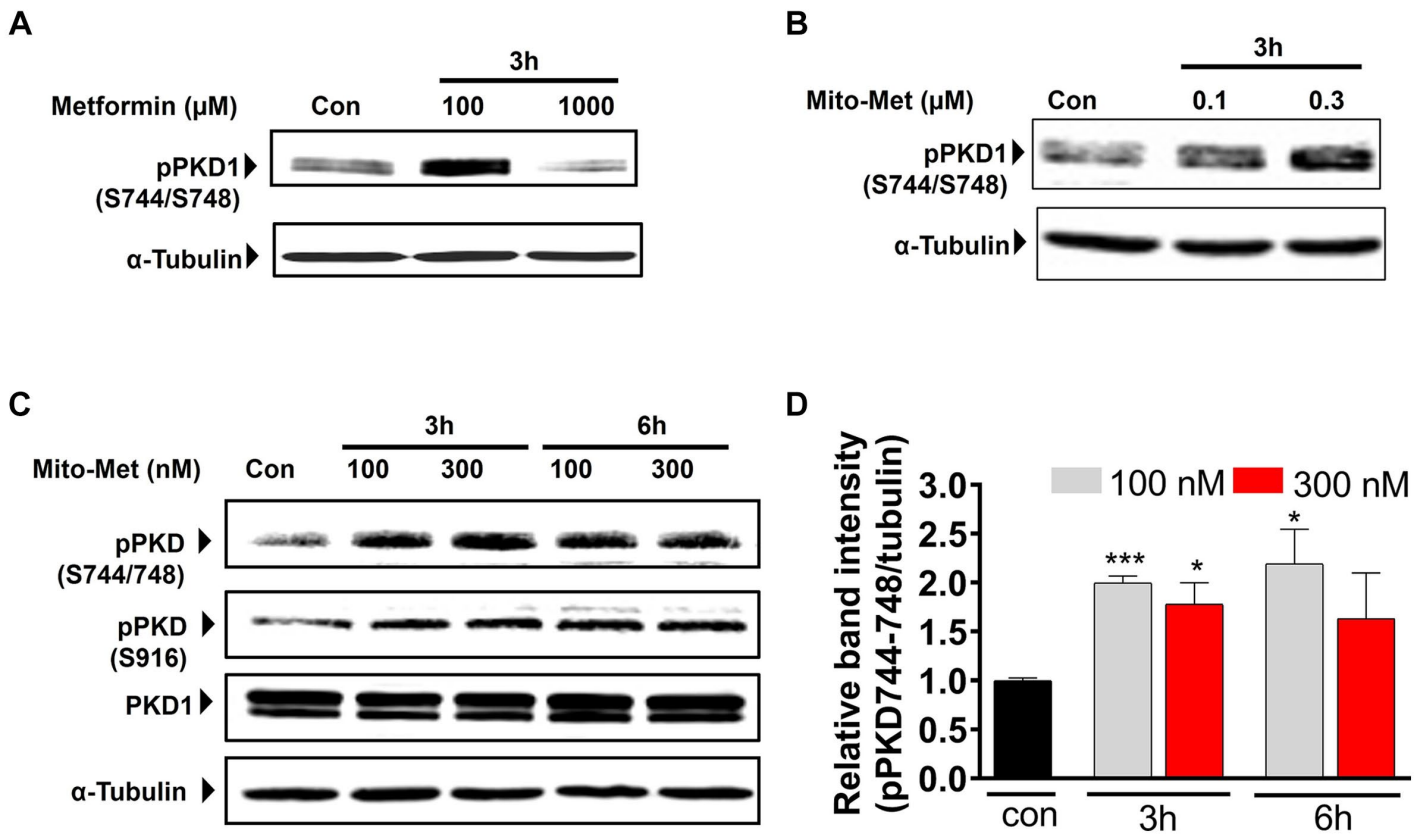
Activation of PKD1 by Mito-Met in N27 cells. **(A)** N27 dopaminergic neuronal cells were treated with metformin (100 and 1,000 μM) for 3 h. **(B)** N27 cells were treated with Mito-Met (100 and 300 nM) for 3 h. Cell lysates were prepared and subjected to Western blot analysis. Representative immunoblots of PKD1 S744/748 phosphorylation are shown. **(C)** N27 cells were treated with 100 and 300 nM Mito-Met for 3 and 6 h. Cell lysates were prepared and subjected to Western blot analysis. Representative immunoblots of total PKD1, PKD1 S744/748, and S916 phosphorylation are shown. **(D)** The graph represents the densitometric analysis of phospho-PKD1 S744/748 levels in **(C)**. Results are the mean ± SEM of at least three independent experiments (^*^p ≤ 0.05; ^***^p < 0.001).

**Figure 2:**
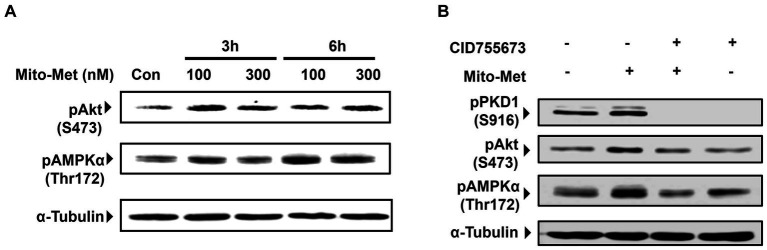
Activation of Akt and AMPK by Mito-Met in N27 cells. **(A)** N27 cells were treated with 100 and 300 nM Mito-Met for 3 and 6 h. Cell lysates were prepared and phospho-Akt (S473) and phospho-AMPKα (Thr172) levels were determined by Western blot analysis. **(B)** N27 cells were pretreated with 50 μM PKD1 inhibitor CID755673 for 1 h and then cotreated with 100 nM Mito-Met for 3 h. Cell lysates were prepared and subjected to Western blot analyses of phospho-PKD1 (S916), phospho-Akt (S473), and phospho-AMPKα (Thr172).

To evaluate our hypothesis that Mito-Met is more potent than its parent compound metformin in providing neuroprotection, we wanted to compare the effects of metformin and Mito-Met on the activation of pro-survival kinase PKD1, reported by our group to have neuroprotective effects against oxidative stress-induced cell death in DAergic neurons (Asaithambi et al., 2011, 2014). We first treated N27 DAergic neuronal cells with the widely accepted dose range of metformin (100 and 1,000 μM) and Mito-Met (100 and 300 nM) for 3 h and checked the phosphorylation of PKD1. We selected Mito-Met concentrations of 100 nM and 300 nM as optimal doses after performing a preliminary dose–response study ranging from 10 nM to 1,000 nM. Also, this dose range of mitochondria-targeted compounds has been shown to greatly increase the mitochondrial concentration (Zielonka et al., 2017; Kalyanaraman et al., 2018). As evident from Figures 1A,B, although both metformin and Mito-Met induced the phosphorylation of PKD1 activation loop (S744/748), Mito-Met was more than 300-fold more efficacious than metformin in activating PKD1. To determine the time course response, we next treated N27 DAergic cells with 100 and 300 nM Mito-Met for 3 h and 6 h and measured both the PKD1 activation loop phosphorylation and the C-terminal phosphorylation (S916). As shown in Figures 1C,D, Mito-Met significantly increased the phosphorylation of both S744/748 and S916 of PKD1 at both 3 and 6 h time points. However, expression of total PKD1 was not affected by Mito-Met treatment (Figure 1C). Following the observation that Mito-Met enhances PKD1 activation, we wanted to determine whether Mito-Met can also increase the activation of Akt because Akt has been previously shown to have neuroprotective effects in cell culture and animal models of PD (Ries et al., 2006; Gunjima et al., 2014). Immunoblot analysis revealed that treatment of N27 cells with Mito-Met (100 nM and 300 nM) for 3 and 6 h increased Akt phosphorylation (Figure 2A). To further explore the potential neuroprotective mechanism of Mito-Met, we examined whether Mito-Met can activate AMPK in DAergic neuronal cells since metformin, the parent compound of Mito-Met, is known to activate AMPK in different cell types, including neurons (Ouyang et al., 2011; Stephenne et al., 2011; Hou et al., 2015). AMPK has also been reported to exert a neuroprotective effect against DAergic neurodegeneration (Ng et al., 2012). To determine AMPK activation, we used an antibody directed against phosphorylated Thr172 within the catalytic subunit (alpha) of AMPK. As shown in Figure 2A, Mito-Met treatment increased AMPK Thr172 phosphorylation in N27 cells. Next, we reasoned that PKD1 might lie upstream of Akt and AMPK kinase during Mito-Met treatment. To test this hypothesis, N27 cells were pretreated with a pharmacological inhibitor of PKD1 (CID755673) for 1 h and then cotreated with CID755673 (50 μM) and Mito-Met (100 nM) for 3 h. Interestingly, treatment with CID755673 blocked the Mito-Met-induced activation of both Akt and AMPK kinase (Figure 2B). Collectively, these data suggest that Mito-Met can effectively activate PKD1 and its potential downstream neuroprotective effectors, Akt and AMPK kinase signaling, in DAergic cells.

### Mito-Met stimulates mitochondrial biogenesis and bioenergetics capacity in DAergic neuronal cells

**Figure 3:**
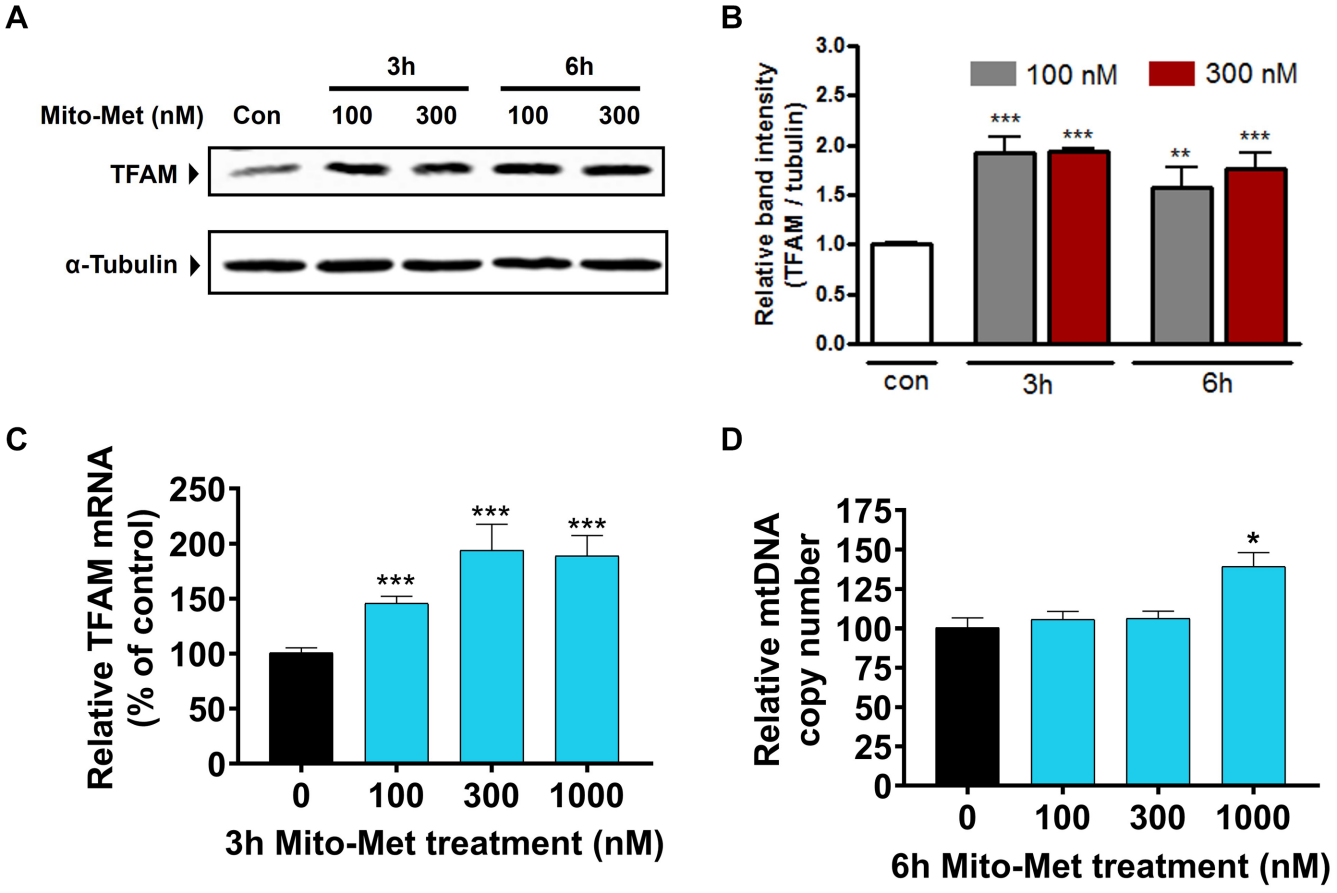
Mito-Met increases TFAM expression in N27 cells. **(A)** N27 cells were treated with 100 and 300 nM Mito-Met for 3 and 6 h. Cell lysates were prepared and TFAM levels were determined by Western blot analysis. **(B)** The graph represents the densitometric analysis of TFAM protein levels normalized to α-tubulin. **(C)** N27 cells were treated with Mito-Met (100–1,000 nM) for 3 h. Real-time RT-PCR analysis of TFAM mRNA level was performed. 18S rRNA served as internal control. **(D)** N27 cells were treated with Mito-Met (100–1,000 nM) for 6 h. Genomic DNA was isolated and mtDNA content was determined by quantitative PCR with SYBR green. 10 ng nuclear DNA and 1 ng mitochondrial DNA were amplified using the primers for mitochondrial ND1 and nuclear β-actin genes. Results are the mean ± SEM of three independent experiments (^*^p ≤ 0.05; ^**^p < 0.01; ^***^p < 0.001; between the control and Mito-Met-treated samples).

**Figure 4:**
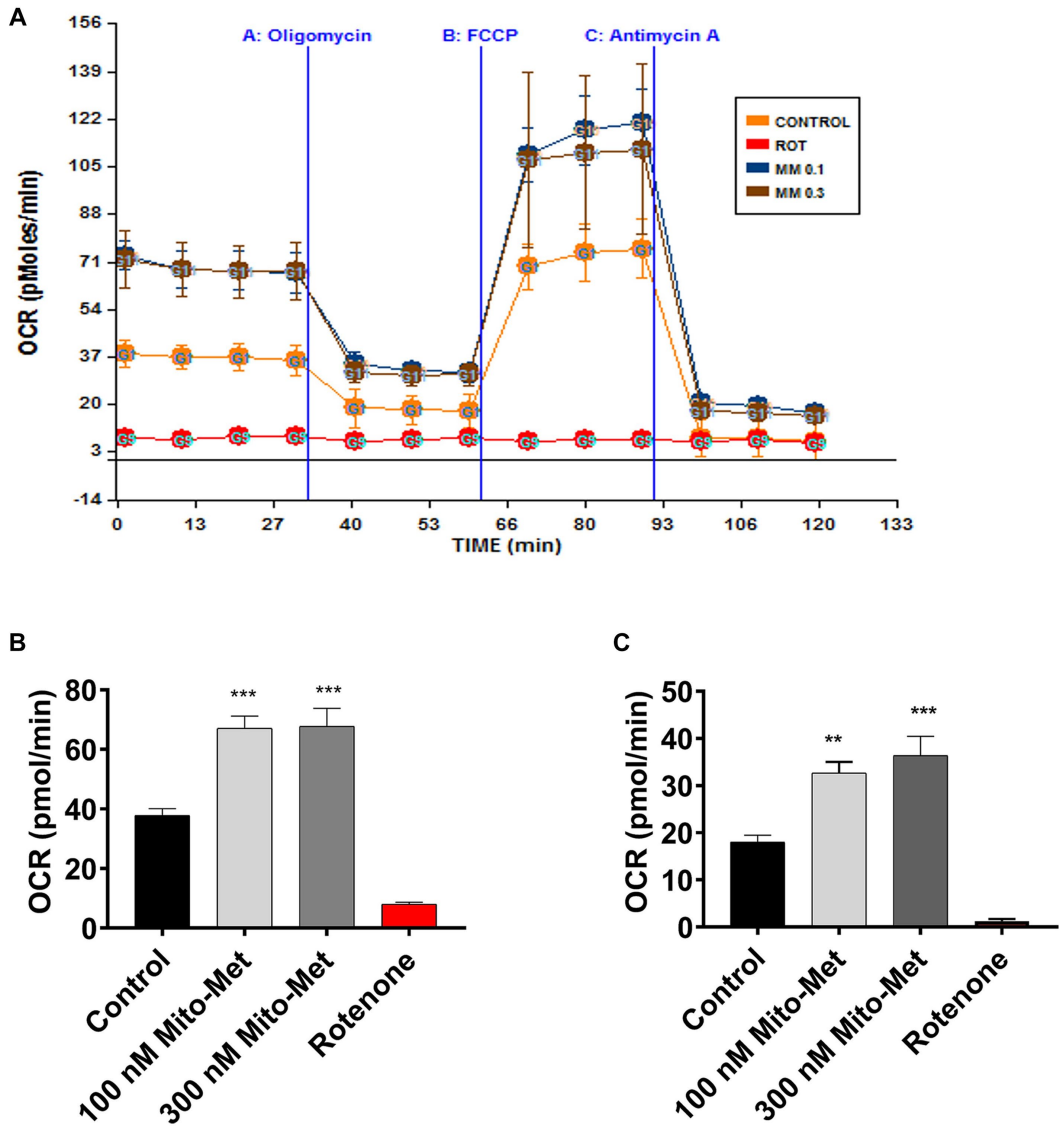
Mito-Met increases mitochondrial bioenergetics capacity. **(A–C)** N27 dopaminergic neuronal cells were treated with 100 and 300 nM Mito-Met for 3 h. Rotenone (1 μM) was included as a positive control. N27 cell-containing culture plates were loaded into the Seahorse XF96 analyzer for the OCR measurement. Mitochondrial dynamics were measured using the sequential injection of oligomycin A (1 μg/mL), FCCP (1 μM), and antimycin A (10 μM) **(A)**. Basal OCR **(B)** and ATP-linked respiration **(C)** were calculated from the output OCR values. Values represent the means ± SEM of four replicates (^**^p < 0.01; ^***^p < 0.001; between the control and Mito-Met-treated samples).

After observing that Mito-Met can enhance the activation of PKD1, we investigated whether Mito-Met stimulates mitochondrial biogenesis. We first investigated the effect of Mito-Met on TFAM expression. TFAM is a transcription factor that is required for mtDNA replication and transcription to regulate mitochondrial biogenesis (Virbasius and Scarpulla, 1994; Scarpulla, 2008). N27 cells were treated with 100 and 300 nM Mito-Met for 3 and 6 h and TFAM levels were determined by Western blot analysis. As seen in Figures 3A,B, the expression of TFAM protein was significantly increased in N27 cells treated with Mito-Met. Next, we measured TFAM mRNA expression using real-time RT-PCR at 3 h time point. Exposure of N27 cells to Mito-Met (100–1,000 nM) significantly increased TFAM mRNA expression (Figure 3C). We also determined mtDNA copy number using mitochondrial ND1 and nuclear β-actin genes and found that Mito-Met treatment (1,000 nM) significantly increased mtDNA content in N27 cells (Figure 3D). Furthermore, we evaluated the effect of Mito-Met on cellular bioenergetics in DAergic neuronal cells. N27 cells were treated with 100 and 300 nM Mito-Met for 3 h and the oxygen consumption rates (OCR) were measured using a Seahorse XF96 analyzer. Interestingly, we observed significant increases in basal OCR and ATP-linked respiration in Mito-Met-treated N27 cells, while rotenone, a well-known mitochondrial complex I inhibitor, almost completely blocked mitochondrial respiration (Figures 4A–C). These results suggest that Mito-Met can stimulate mitochondrial biogenesis and oxidative phosphorylation in DAergic cells.

### Mito-Met protects against MPP^+^-induced mitochondrial damage and neurotoxicity

**Figure 5:**
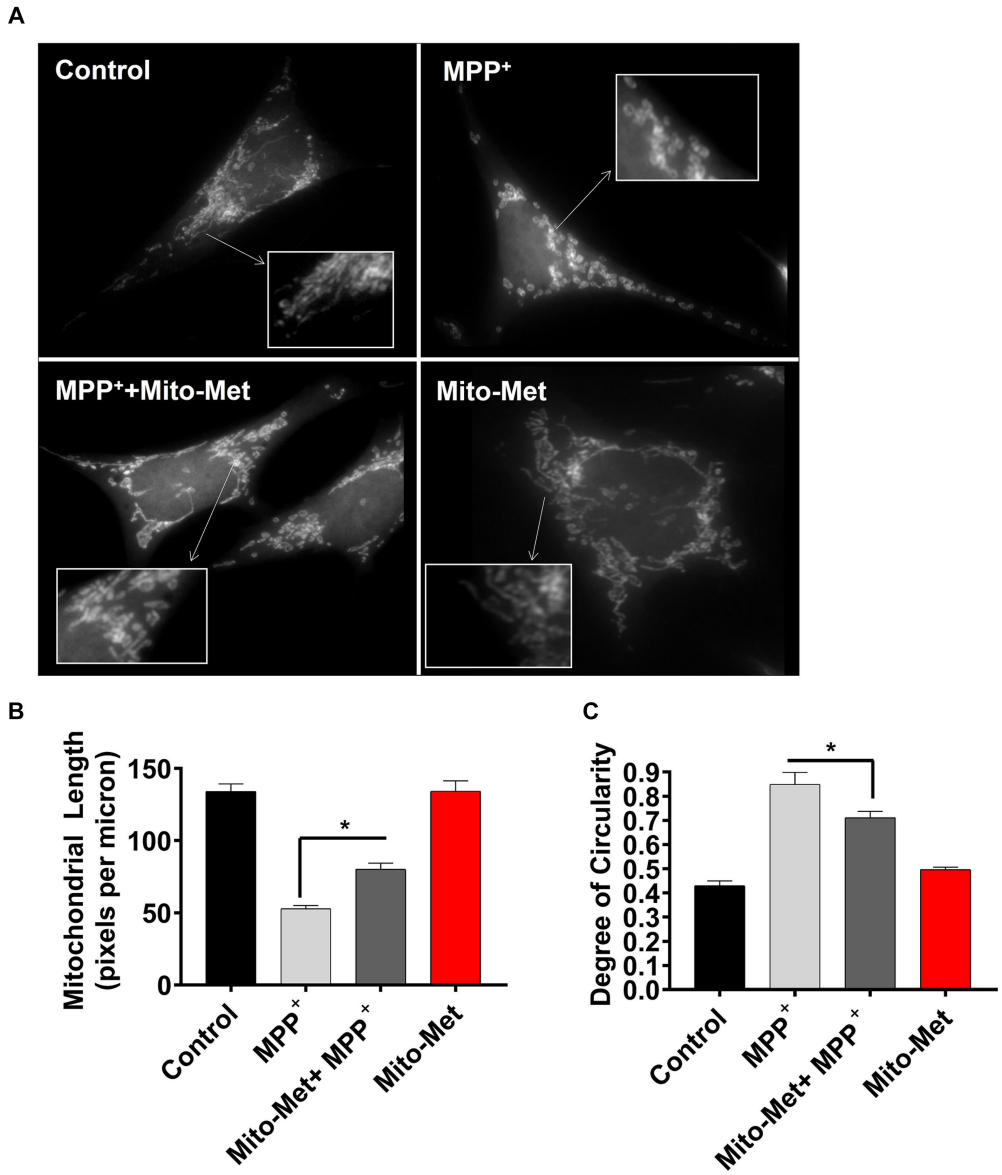
Mito-Met reduces MPP^+^-induced mitochondrial fragmentation. **(A–C)** N27 cells were pretreated with 300 nM Mito-Met for 1 h, and then cotreated with 300 μM MPP^+^ for 16 h. Cells were stained with the MitoTracker red dye. Images were taken at a magnification of 60X **(A)**. Mitochondrial length **(B)** and degree of circularity **(C)** were quantified using ImageJ. Values represent the means ± SEM of two independent experiments performed in sextuplicate (^*^p ≤ 0.05; between the Mito-Met-pretreated and MPP^+^ (alone)-treated groups).

**Figure 6:**
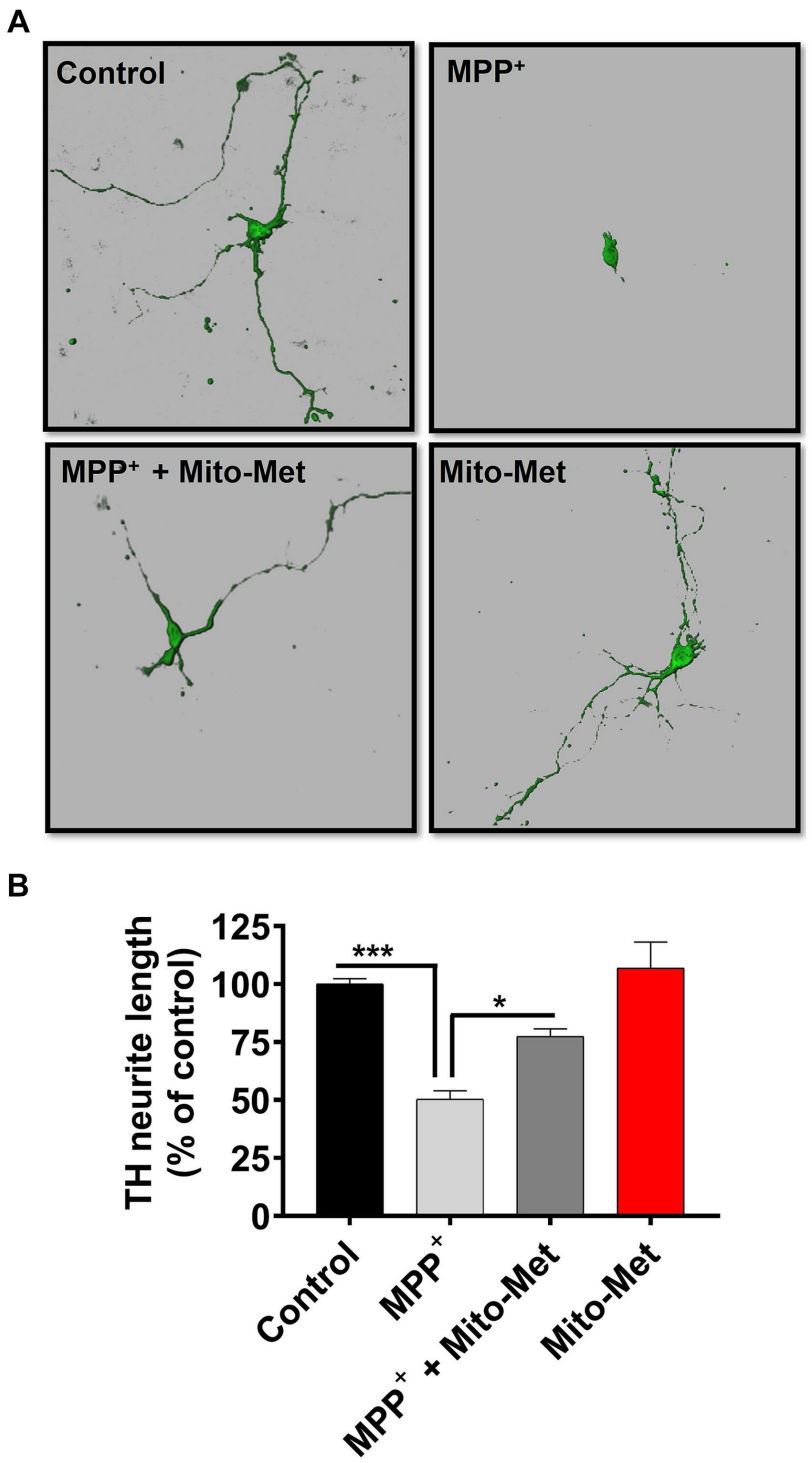
Mito-Met protects against MPP^+^-induced neurotoxicity in primary neurons. **(A,B)** The primary mesencephalic neurons were treated with 10 μM MPP^+^ in the presence or absence of Mito-Met (100 nM) for 24 h and immunostained with an TH antibody. Images were taken at 60X magnification using the Leica confocal microscope and 3D image reconstruction was performed using IMARIS software **(A)**. The length of TH-positive neuronal processes in primary dopaminergic neurons from each coverslip was measured using MetaMorph software **(B)**. The experiments were performed in sextuplicate (^*^p ≤ 0.05; ^***^p < 0.001).

To further examine the potential neuroprotective influence of Mito-Met on mitochondrial function, we examined whether Mito-Met can reduce MPP^+^-induced mitochondrial damage. N27 DAergic cells were pretreated with 300 nM Mito-Met for 1 h and then cotreated with Mito-Met and MPP^+^ (300 μM) for 16 h and stained with MitoTracker to visualize mitochondrial morphology. Expectedly, MPP^+^ treatment strongly induced mitochondrial fragmentation (Figure 5A); however, Mito-Met pre- and co-treatment significantly ameliorated mitochondrial morphology in MPP^+^-treated cells as evidenced by improved mitochondrial length and circularity (Figures 5B,C). Next, we tested the protective effect of Mito-Met in primary DAergic neurons cultured from E14 mouse embryos. The primary nigral neurons were treated with 10 μM MPP^+^ in the presence or absence of Mito-Met (100 nM) for 24 h and immunostained with a TH antibody. To evaluate whether Mito-Met treatment protects against MPP^+^-induced DAergic neurodegeneration, we measured the length of DAergic neurites. The immunocytochemical analysis revealed that Mito-Met treatment significantly protected against MPP^+^-induced neurotoxicity, as evidenced by a significant increase in the length of neuronal processes in primary nigral cultures cotreated with MPP^+^ and Mito-Met compared with cultures treated with MPP^+^ alone (Figures 6A,B). Together, these data clearly demonstrate that Mito-Met can effectively protect DAergic neurons from MPP^+^-induced toxicity.

### Mito-Met improves motor deficits and attenuates striatal DA depletion in MitoPark mice

**Figure 7:**
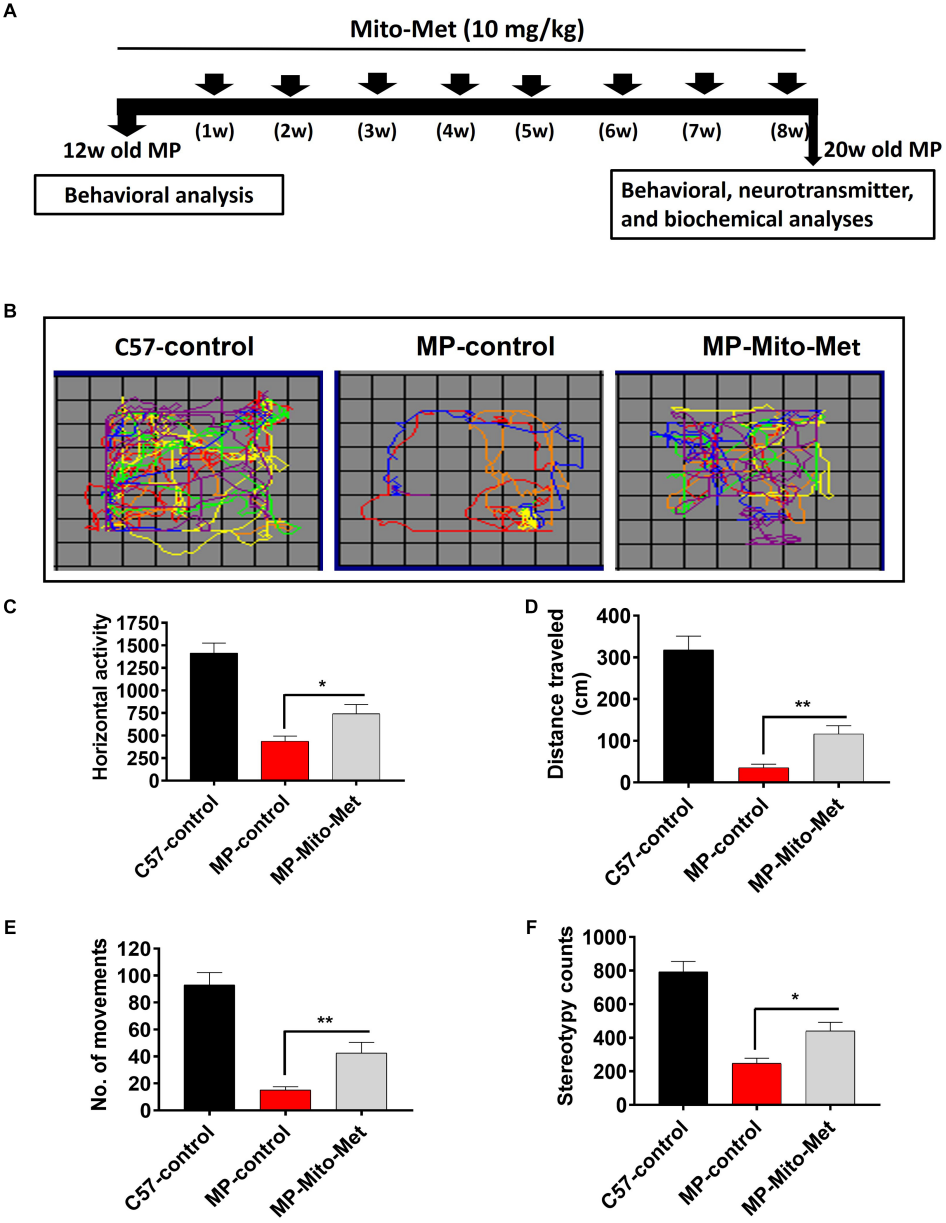
Mito-Met improves motor deficits in MitoPark mice. **(A)** Treatment schedule of MitoPark mice with Mito-Met. 12-week-old MitoPark mice were treated with either saline or Mito-Met (10 mg/kg) via oral gavage three times per week for 8 weeks. The locomotor activities were measured using a VersaMax system 1 day prior to sacrifice. Moving track of mice **(B)**, horizontal activity **(C)**, total distance traveled **(D)**, number of movements **(E)**, and stereotypy counts **(F)**. Values represent the means ± SEM of six mice per group (^*^p ≤ 0.05; ^**^p < 0.01).

**Figure 8:**
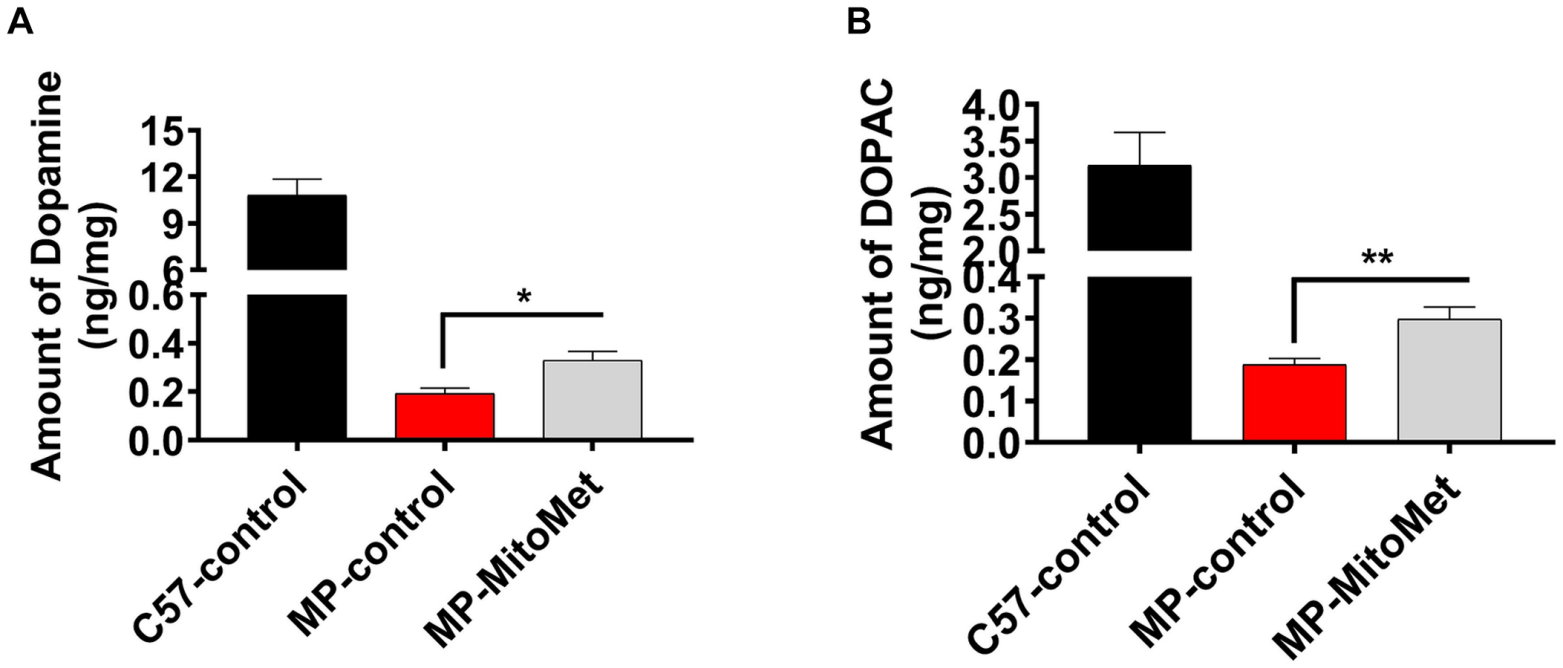
Mito-Met attenuates striatal dopamine depletion in MitoPark mice. **(A,B)** 12-week-old MitoPark mice were treated with either saline or Mito-Met (10 mg/kg) via oral gavage three times per week for 8 week. Mice were sacrificed 1 day after the last Mito-Met treatment and dopamine **(A)** and DOPAC **(B)** levels were measured from striatal tissues by HPLC analysis. Results are the means ± SEM of five mice per group (^*^p ≤ 0.05; ^**^p < 0.01).

The results of our *in vitro* experiments demonstrated that Mito-Met affords neuroprotection against DAergic neurotoxicity. Here, we wanted to test the neuroprotective efficacy of Mito-Met in the MitoPark mouse model of PD. As seen in Figure 7A, MitoPark and control mice were treated with either saline or Mito-Met (10 mg/kg) three times per week by oral gavage for 8 weeks. One day prior to sacrifice, mice were analyzed for their locomotor activities. MitoPark mice develop progressive motor deficits from 12 weeks of age onwards (Ekstrand et al., 2007). As expected, 20-week-old MitoPark mice had a dramatic reduction in total movement (Figure 7B). Notably, improved locomotion was observed in the MitoPark mice-treated with Mito-Met. Mito-Met treatment reversed the reduction in horizontal activity (Figure 7C), total distance traveled (Figure 7D), number of movements (Figure 7E), and stereotypy counts (Figure 7F) of MitoPark mice. Moreover, we also determined whether Mito-Met treatment attenuates striatal DA depletion in MitoPark mice. Mice were sacrificed 1 day after the last Mito-Met treatment and striatal tissues were processed for HPLC analysis of DA. Neurochemical analysis revealed that the DA and DOPAC levels were significantly higher in the MitoPark mice-treated with Mito-Met than the MitoPark mice-treated with saline (Figures 8A,B). Collectively, these results suggest that Mito-Met treatment can improve motor deficits and at least partially prevent striatal DA depletion in MitoPark mice.

## Discussion

The findings of this study provide evidence that a mitochondria-targeted analog of metformin, Mito-Met, exerts neuroprotective effects in cell culture and MitoPark mouse models of PD. Our results revealed that Mito-Met can effectively activate PKD1-mediated neuroprotective signaling. We also demonstrate that Mito-Met enhances mitochondrial biogenesis and bioenergetics capacity. In addition, Mito-Met protects against MPP^+^ neurotoxicity in a cell model of PD. Finally, and perhaps most importantly, Mito-Met treatment has neuroprotective effects against behavioral deficits and striatal DA depletion in the MitoPark transgenic mouse model of PD.

Metformin has been used for several decades to treat diabetic patients. Besides its anti-diabetic effect, metformin has been shown to have neuroprotective effects in experimental stroke (Li et al., 2010), against ethanol-induced neuronal apoptosis (Ullah et al., 2012), alpha-synuclein neurotoxicity (Dulovic et al., 2014) and MPTP- and rotenone-induced DAergic neurodegeneration (Patil et al., 2014; Lu et al., 2016; El-Ghaiesh et al., 2020; Wang et al., 2020). In this study, we synthesized Mito-Met, a mitochondria-targeted (TPP^+^ conjugated) analog of metformin. Mitochondria-targeted compounds have been reported to show neuroprotective effects in experimental models of PD by virtue of their reduced toxicity and increased accumulation inside the mitochondria (Yang et al., 2009; Ghosh et al., 2010; Solesio et al., 2013; Kalyanaraman, 2020). Here, we first examined whether Mito-Met can activate the PKD1 pro-survival signaling in DAergic neuronal cells. Previous studies from our group suggest that positive modulators of PKD1 signaling could serve as potential neuroprotective agents for the treatment of PD (Asaithambi et al., 2011, 2014; Ay et al., 2015; Bejanin et al., 2017). Our results demonstrated that Mito-Met at nanomolar concentrations can activate PKD1, whereas micromolar concentrations of metformin are needed to activate PKD1 in DAergic neuronal cells (Figures 1A,B), suggesting Mito-Met is much more potent than its parent compound metformin. To identify molecular mechanisms governing the potential neuroprotective effect of Mito-Met, we also explored Akt and AMPK cell survival signaling and observed increased phosphorylation of Akt and AMPK in Mito-Met-treated cells (Figure 2A). Akt has been previously implicated in the survival of DAergic neurons (Ries et al., 2006; Gunjima et al., 2014). AMPK is a well-known target of metformin in different cell types, including non-neuronal and neuronal cells (Ouyang et al., 2011; Stephenne et al., 2011; Hou et al., 2015) and has been reported to protect against DAergic neurodegeneration in cell culture and Drosophila models of PD (Ng et al., 2012; Dulovic et al., 2014). Moreover, our studies using a pharmacological inhibitor of PKD1, CID755673, revealed that PKD1 appears to act upstream of Akt and AMPK kinases during the Mito-Met treatment of DAergic cells (Figure 2B). However, more mechanistic studies using both genetic and pharmacological approaches are required to fully identify the relationship between these kinases in DAergic neurons. It’s worth mentioning that we have previously utilized a different PKD1 inhibitor, kbNB-14270, and observed its capacity to inhibit PKD1/Akt activation induced by quercetin in DAergic neuronal cells (Ay et al., 2017). However, we recently found that CID755673 exhibits a higher degree of specificity as an inhibitor of PKD1 compared to kbNB-14270. Also, our unpublished data revealed that CID755673 is able to completely inhibit PKD1 kinase activity. Thus, we focused our investigation solely on CID755673 in this study.

Metformin has been previously shown to inhibit mitochondrial complex I activity in both hepatoma and cancer cells (Owen et al., 2000; Andrzejewski et al., 2014) and the anti-cancer effect of metformin is attributed to its inhibitory effect on mitochondrial complex I (Wheaton et al., 2014). However, the notion that metformin inhibits complex I is controversial because preservation of complex I activity has been reported in metformin-treated mice (Martin-Montalvo et al., 2013) and in the plasma of metformin-treated diabetic patients (Larsen et al., 2012). Because of these conflicting reports, we wanted to determine whether Mito-Met has any inhibitory effect on mitochondrial function in DAergic cells. Our results revealed that Mito-Met, even at higher concentrations (1 and 10 μM), does not adversely affect mitochondrial function as evidenced by the MitoSOX (Supplementary Figures 1A,B) and aconitase activity (Supplementary Figure 1C) assays. We also evaluated the effect of Mito-Met on mitochondrial biogenesis. Our data show that Mito-Met can increase TFAM expression (Figures 3A–C) and mtDNA copy number (Figure 3D), suggesting Mito-Met can promote mitochondrial biogenesis in DAergic cells. Next, we determined the effect of Mito-Met on mitochondrial respiration. Interestingly, Mito-Met treatment resulted in significant increases in basal OCR (Figure 4B) and ATP-linked respiration (Figure 4C) in DAergic neuronal cells. Together, our results indicate that our mitochondria-targeted metformin analog (Mito-Met) can improve mitochondrial biogenesis and function in DAergic cells.

After observing enhanced activation of survival kinases and mitochondrial function in Mito-Met-treated cells, we evaluated the potential neuroprotective effect of Mito-Met against MPP^+^-induced neurotoxicity. We first assessed the effect of Mito-Met pretreatment on mitochondrial morphology and integrity in MPP^+^-treated N27 cells and observed that Mito-Met significantly reduced mitochondrial fragmentation induced by MPP^+^ (Figures 5A,B). Of note, there was no change in mitochondrial morphology in N27 cells treated with Mito-Met alone. Next, we determined the protective effect of Mito-Met against MPP^+^-induced DAergic neurodegeneration in primary mesencephalic cultures. Importantly, co-treatment of primary mesencephalic neurons with Mito-Met significantly protected against MPP^+^-induced TH-positive neurite loss (Figures 6A,B), further supporting the neuroprotective effect of Mito-Met in DAergic cells.

To test the neuroprotective efficacy of Mito-Met *in vivo*, we used the MitoPark transgenic mouse model of PD. This transgenic mouse model of mitochondrial dysfunction recapitulates several features of human PD, including adult onset, progressive DAergic neurodegeneration and motor deficits (Ekstrand et al., 2007; Galter et al., 2010). Since MitoPark mice begin to show motor deficits from 12 weeks of age onwards, we treated 12-week-old MitoPark mice with either saline or Mito-Met for 8 weeks. Our results revealed that Mito-Met significantly reversed behavioral deficits in MitoPark mice (Figures 7B–F). Furthermore, higher levels of DA and DOPAC were observed in the Mito-Met-treated MitoPark mice compared with the MitoPark mice treated with saline, suggesting Mito-Met can protect against DAergic neurodegeneration in MitoPark mice. It should be noted that, compared to the significant behavioral improvements, the neurochemical outcomes appear to be relatively moderate. As seen in human PD cases, pronounced behavioral deficits occur in our MitoPark transgenic PD model only after a 60–70% loss of striatal DA levels. Thus, it is possible that even a moderate restoration of striatal DA can significantly improve behavioral outcomes. This phenomenon can also be attributed to the complex interplay of multiple neural pathways and neurotransmitter systems influencing locomotor outcomes in PD models, beyond the sole consideration of DA levels (Dubuc et al., 2023). The restoration of DA levels in the striatum by Mito-Met represents only one facet of its overall neuroprotective effects. Furthermore, this DA effect can extend to other areas and circuits of the brain responsible for motor learning behavior, such as the hippocampus, amygdala, and frontal cortex. As the striatum integrates information from these diverse regions, Mito-Met may promote mitochondria-dependent neuroprotective pathways such as mitochondrial biogenesis in neurons associated with motor learning circuits (Cataldi et al., 2022), leading to the observed significant improvements in locomotor behavior. Additionally, blood chemistry analysis revealed no adverse effects in Mito-Met-treated mice (Supplementary Table 1).

Metformin is generally recognized as a safe and effective medication for managing conditions, including diabetes. Like many medications, it can have multiple off-target effects in addition to its primary therapeutic benefits. Some of these off-target effects include gastrointestinal disturbances, microbiota changes, decreased folate levels, vitamin B12 deficiency, and the rare occurrence of lactic acidosis (Xu et al., 2013; Blough et al., 2015; Bonnet and Scheen, 2017; Silamiķele et al., 2021). However, when it comes to Mito-Met, we find no evidence regarding its off-target effects. Additional research is needed to comprehensively understand the full safety profile of Mito-Met.

In conclusion, our data demonstrate that a mitochondria-targeted metformin analog (Mito-Met) activates PKD1-mediated neuroprotective signaling and enhances mitochondrial function. Importantly, Mito-Met can protect against MPP^+^-induced neurotoxicity in cell culture models of PD. Additionally, Mito-Met treatment can reverse behavioral deficits and attenuate DA depletion in the MitoPark animal model of PD. Overall, our results suggest that Mito-Met is a potential neuroprotective agent that deserves further preclinical evaluations for the treatment of PD.

## Materials and methods

### Chemicals and reagents

1-Methyl-4-phenyl pyridinium iodide (MPP^+^ iodide) and anti-β-actin antibody were purchased from Sigma-Aldrich (St. Lois, MO). The Bradford protein assay kit was purchased from Bio-Rad. Antibodies against PKD1, α-tubulin, and mtTFA were purchased from Santa Cruz Biotechnology (Santa Cruz, CA). Anti-phospho PKD1 (p-S744/748 and p-S916), phospho-Akt (p-S473), total Akt, phospho-AMPKα (p-Thr172) antibodies were obtained from Cell Signaling Technology (Danvers, MA). Anti-TH antibody was purchased from Millipore (Billerica, MA). The PKD1 inhibitor CID755673 was purchased from Tocris Bioscience (Ellisville, MO). Alexa 680-conjugated anti-mouse secondary antibody, MitoTracker Red FM, and all cell culture reagents were obtained from Invitrogen (Carlsbad, CA). IRDye 800-conjugated anti-rabbit secondary was purchased from Rockland Labs (Gilbertsville, PA).

### Cell cultures

The rat DAergic N27 cell line was a kind gift from Dr. Kedar N. Prasad (University of Colorado Health Sciences Center, Denver, CO) and cultured as described previously (Kaul et al., 2003). Briefly, N27 cells were grown in RPMI 1640 medium supplemented with 10% fetal bovine serum, 2 mM L-glutamine, 50 units penicillin, and 50 μg/mL streptomycin and maintained at 37°C in a 5% CO2 atmosphere. We also used the primary mesencephalic neuronal culture to assess the protective effect of Mito-Met against MPP^+^-induced TH-positive neurite loss. The primary mesencephalic culture was prepared from the ventral mesencephalon of gestational 14-d-old mouse embryos as described previously (Zhang et al., 2007). Briefly, the mesencephalic tissues from E14 mouse embryos were dissected and maintained in ice cold Ca^2+^-free Hank’s balanced salt solution, and then dissociated in Hank’s balanced salt solution containing trypsin-0.25% EDTA for 30 min at 37°C. Cultures were maintained in Neurobasal medium supplemented with B27. Half of the culture medium was replaced every 2 d. Approximately 6- to 7-d-old cultures were used for experiments. Primary mesencephalic neuronal cells were treated with 10 μM MPP^+^ in the presence or absence of Mito-Met (100–300 nM) for 24 h.

### Quantitative real-time RT-PCR

Total RNA was isolated from cells using the Absolutely RNA Miniprep Kit (Stratagene, La Jolla, CA), and 1–2 μg RNA was used to generate cDNA using the High-Capacity cDNA Reverse Transcription Kit (Applied Biosystems). Real-time PCR was performed on an Mx300P QPCR system (Stratagene) using the RT^2^ SYBR Green qPCR Master Mix Kit (Qiagen) and QuantiTect Primer Assay Kit (Qiagen). All RT-qPCR reactions were performed in triplicate and normalized to the β-actin housekeeping gene. The primer sequences and PCR conditions are available upon request. Dissociation curves were run to verify the singularity of the PCR product. The data were analyzed using the comparative threshold cycle (Ct) method.

### Quantitative real-time PCR analysis of mtDNA content

N27 cells were plated in 6-well plates at a density of 2 × 10^5^ cells/well. Twenty-four hours later, cells were treated with 0.1–1 μM Mito-Met for 24 h. Genomic DNA was isolated using the DNeasy Blood and Tissue Kit (Qiagen, Valencia, CA) according to the manufacturer’s protocol, and mtDNA content was determined by quantitative PCR with SYBR green. 10 ng nuclear DNA and 1 ng mitochondrial DNA were amplified using the primers for mitochondrial ND1 and nuclear β-actin genes. Reactions were performed using an Mx300P QPCR system (Stratagene).

### Western blot analysis

After treatments, cells or brain tissues were collected and resuspended in a modified RIPA buffer containing protease and phosphatase inhibitors. Briefly, lysates containing equal amounts of protein were loaded in each lane and separated on a 10–15% SDS-PAGE gel and transferred onto a nitrocellulose membrane (Bio-Rad). The membranes were blocked in LI-COR blocking buffer for 1 h at room temperature (RT) and then incubated overnight at 4°C with the corresponding primary antibodies. Western blot was performed using IRDye 800 anti-rabbit, Alexa Fluor 680 goat anti-mouse, and Alexa Fluor 680 donkey anti-goat secondary antibodies. Western blot images were captured and analyzed with an Odyssey Infrared Imaging System (LI-COR, Lincoln, NE).

### Immunocytochemistry and quantification of TH-positive neuronal processes

Primary mesencephalic DAergic neurons were fixed with 4% paraformaldehyde for 30 min at RT. After washing, cells were permeabilized with blocking agent (2% bovine serum albumin, 0.5% Triton X-100, and 0.05% Tween-20 in PBS) for 1 h. Cells were then incubated with an anti-TH antibody (1:1,200, Millipore) at 4°C overnight. Fluorescently conjugated secondary antibody (Alexa Fluor 488-conjugated anti-mouse antibody, 1:2,000) was used followed by incubation with 10 μg/mL Hoechst 33342 for 5 min at RT to stain the nucleus. Cover slips were mounted on glass slides and cells were visualized by Z-Stack imaging with the Leica confocal microscope. 3D image reconstruction was performed using IMARIS software. The length of TH-positive neuronal processes in primary DAergic neurons from each coverslip was measured using MetaMorph software (Molecular Devices) as described previously (Ghosh et al., 2013; Charli et al., 2015). For quantification of neuronal processes, pictures were taken at 60X magnification. TH-positive neuronal processes were counted in six individual cultures for each treatment.

### MitoTracker staining and analysis of mitochondrial morphology

N27 cells were stained with MitoTracker Red FM (200 nM) for 30 min at 37°C, and then fixed with 4% paraformaldehyde for 30 min at RT. After washing, cells were permeabilized with 0.5% Triton X-100 in PBS for 5 min at RT, as described previously (Charli et al., 2015). Cover slips were mounted on glass slides and imaged through a Nikon TE2000 microscope with a SPOT color digital camera (Diagnostic Instruments, Sterling Heights, MI). Quantification of mitochondrial parameters such as mitochondrial length and degree of circularity was performed using a macro text file plug-in for ImageJ (Dagda et al., 2009).

### Measurement of mitochondrial oxygen consumption

The Seahorse XF96 Extracellular Flux Analyzer (Seahorse Bioscience, North Billerica, MA) was used to measure oxygen consumption rates (OCR) in N27 cells. Briefly, N27 cells were seeded at 10,000 cells/well in a Seahorse Bioscience polystyrene microplate. Twenty-four hours later, cells were treated with 100 and 300 nM Mito-Met for 3 h. After the treatment, cells were changed to assay media and the N27 cell-containing culture plates were loaded into the Seahorse instrument to collect baseline OCR measurements. The experimental agents (1 μg/mL oligomycin, 3 μM FCCP, and 10 μM antimycin A) were injected using the included ports on the XF96 cartridges and OCR measurements were collected. Basal OCR, ATP-linked OCR, maximal OCR, and spare respiratory capacity were calculated, as described previously (Dranka et al., 2010).

### Animals and treatment

MitoPark and C57BL/6 mice (12 weeks old) were housed under standard conditions: constant temperature (22 ± 1°C), humidity (relative, 30%), and a 12 h light/dark cycle. Mice were given free access to food and water. Animal care and protocol procedures were approved and supervised by the Institutional Animal Care and Use Committee (IACUC) at Iowa State University (Ames, IA). Mice were treated with either saline or Mito-Met (10 mg/kg) via oral gavage three times weekly for 8 weeks. After the treatments, mice were subjected to behavioral, neurochemical, and biochemical measurements.

### High-performance liquid chromatography analysis

The striatal DA, dihydroxyphenylacetic acid (DOPAC), and homovanillic acid (HVA) levels were quantified using HPLC with electrochemical detection. Samples were prepared and quantified, as described previously (Ghosh et al., 2013). Briefly, neurotransmitters from striatal tissues were extracted in 0.1 M perchloric acid containing 0.05% Na_2_EDTA and 0.1% Na_2_S_2_O_5_ and isoproterenol (internal standard). DA, DOPAC, and HVA were separated isocratically using a C-18 reversed-phase column with a flow rate of 0.6 mL/min. An HPLC system (ESA, Bedford, MA) with an automatic sampler equipped with a refrigerated temperature control was used for these experiments.

### Behavioral measurements

The open field test was performed using an infra-red (IR) activity-tracking system (VersaMax monitor, model RXYZCM-16, and analyzer, model VMAUSB, AccuScan, Columbus, OH). We recorded spontaneous exploratory activity for a 10-min test period after a 2-min acclimatization period. The system quantifies activity by counting and mapping IR beam breaks within a 20 × 20-cm arena. The measures for horizontal activity, number of movements, and stereotypy count (beam breaks) was obtained and plotted. Before test day, animals were acclimatized to the instrument for 10 min for two consecutive days. Data were collected and analyzed using VersaPlot and VersaDat software.

### Statistical analysis

Data analysis was performed using Prism 4.0 software (GraphPad Software, San Diego, CA). Data were first analyzed by using one-way ANOVA and then the Tukey multiple comparison test was used for statistical comparisons. Differences with *p* ≤ 0.05 were considered significant.

## Data availability statement

The original contributions presented in the study are included in the article/Supplementary material, further inquiries can be directed to the corresponding author.

## Ethics statement

The animal study was approved by the Institutional Animal Care and Use Committee (IACUC) at Iowa State University. The study was conducted in accordance with the local legislation and institutional requirements.

## Author contributions

MA: Conceptualization, Data curation, Methodology, Software, Supervision, Validation, Visualization, Writing – original draft, Writing – review & editing. AC: Data curation, Methodology, Validation, Writing – review & editing. ML: Data curation, Methodology, Validation, Writing – review & editing. AJ: Data curation, Methodology, Writing – review & editing. PP: Data curation, Methodology, Writing – review & editing. HJ: Writing – review & editing. VA: Writing – review & editing. BK: Resources, Writing – review & editing. ArK: Funding acquisition, Writing – review & editing. AnK: Conceptualization, Funding acquisition, Project administration, Resources, Supervision, Writing – review & editing.
