# Supplementary material for: Mito-metformin protects against mitochondrial dysfunction and dopaminergic neuronal degeneration by activating upstream PKD1 signaling in cell culture and MitoPark animal models of Parkinson’s disease
Source: Front Neurosci. 2024 Feb 21;18:1356703. doi: 10.3389/fnins.2024.1356703 (PMC10915001; doi:10.3389/fnins.2024.1356703)
Supplement: Supplementary file 1 [file Table_1.DOCX]

Supplementary Material


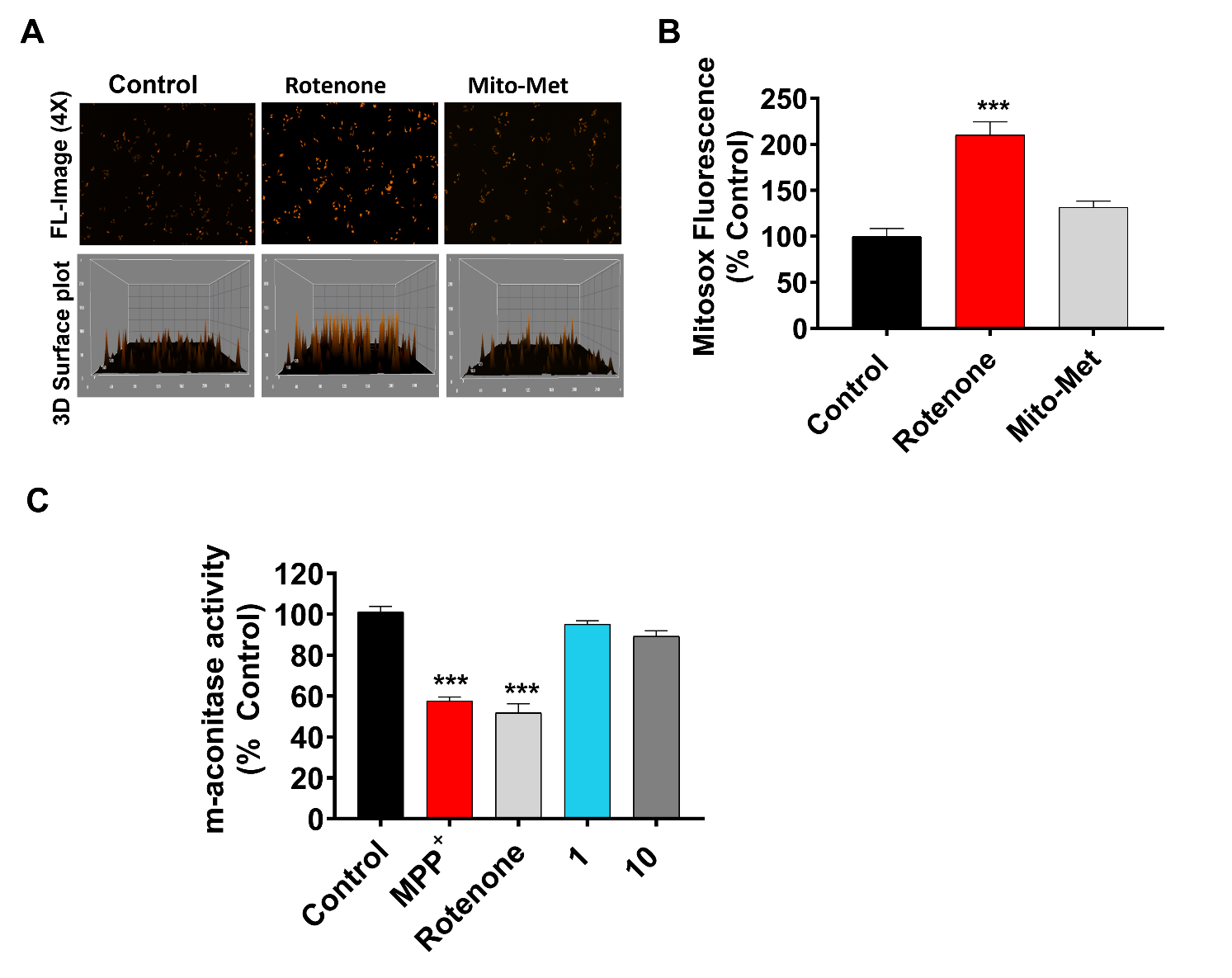


**Supplementary Figure 1.** Mito-Met doesn’t adversely affect mitochondrial function. A-B, N27 cells were treated with 1 µM rotenone and 1 µM Mito-Met for 6 h. Mitochondrial ROS generation was measured using the MitoSOX staining, and fluorescence images were taken at 4X magnification (A). MitoSOX fluorescence was quantified using the Cytation 3 microplate reader (B). C, N27 cells were treated with 300 µM MPP+, 1 µM rotenone, and 1 and 10 µM Mito-Met for 6 h and mitochondrial aconitase activity was measured.


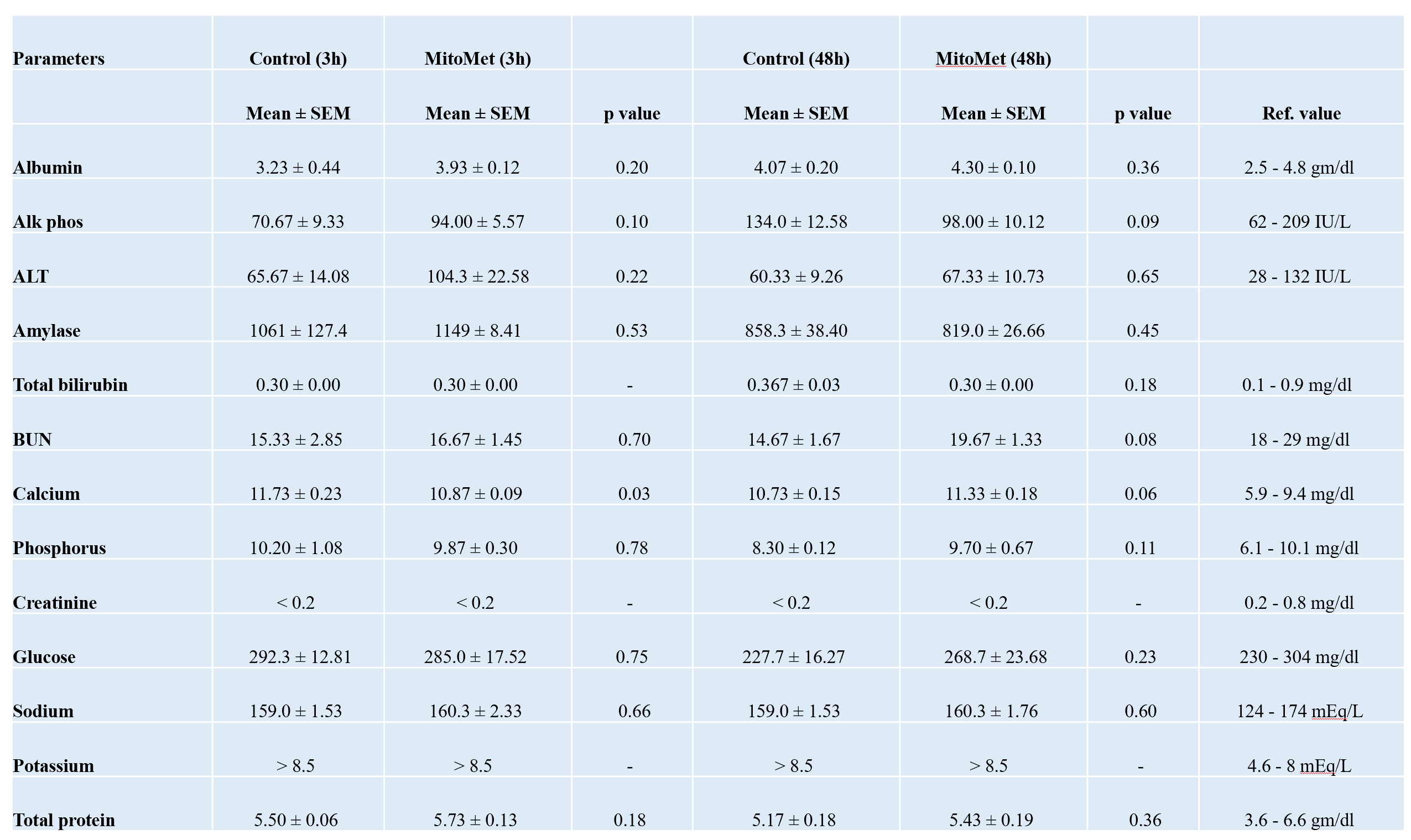


**Supplemental Table 1.** Clinical pathology results for mice (n=3) treated with Mito-Met. Eight- to 10-week-old C57BL/6 mice were treated with Mito-Met (10 mg/kg) via oral gavage for 3 h and 48 h. Blood was drawn from mice by cardiac puncture and sent to the ISU Clinical Pathology Laboratory.
